# Combined effect of graded Thera-Band and scapular stabilization exercises on shoulder adhesive capsulitis post-mastectomy

**DOI:** 10.1007/s00520-023-07641-6

**Published:** 2023-03-16

**Authors:** Nancy H. Aboelnour, FatmaAlzahraa H. Kamel, Maged A. Basha, Alshimaa R. Azab, Islam M. Hewidy, Mohamed Ezzat, Noha M. Kamel

**Affiliations:** 1 Department of Physical Therapy for Surgery, Faculty of Physical Therapy, Cairo University, 7 Ahmed Elzayat St, Bein El sarayat, Dokki, PO 12624 Giza, Egypt; 2 Department of Physical Therapy, College of Medical Rehabilitation, Qassim University, Buraidah, Qassim Saudi Arabia; 3 Department of Physical Therapy, El-Sahel Teaching Hospital, General Organization for Teaching Hospitals and Institutes, Cairo, Egypt; 4 Department of Health and Rehabilitation Sciences, College of Applied Medical Sciences, Prince Sattam Bin Abdulaziz University, Al-Kharj, Saudi Arabia; 5 Department of Physical Therapy for Pediatrics, Faculty of Physical Therapy, Cairo University, Giza, Egypt; 6 Adjunct Faculty at Physical Therapy Department, New York Medical College, Valhalla, NY USA; 7 Department of Physical Therapy for Orthopedics, Faculty of Physical Therapy, Cairo University, Giza, Egypt

**Keywords:** Thera-Band exercises, Scapular stabilization exercises, Mastectomy, Adhesive capsulitis

## Abstract

**Purpose:**

The main aim of the trial was to assess the combined impact of graded Thera-Band strengthening exercises and scapular stabilization exercises on shoulder pain, physical function, and quality of life (QoL) in post-mastectomy adhesive capsulitis (AC).

**Methods:**

Seventy females with unilateral post-mastectomy AC partook in the trial. Participants were subdivided equally into two groups at random. Both groups obtained the traditional physical therapy program; in addition, the intervention group received graded Thera-Band exercises for shoulder muscles and scapular stabilization exercises 5 days a week for 8 weeks. Range of motion (ROM) and muscle power of shoulder were assessed by digital goniometer and handheld dynamometer, respectively. Disability of the Arm, Shoulder, and Hand questionnaire (DASH) was utilized for assessment of shoulder function and visual analogue scale (VAS) for pain measurement while short-form (SF-36) for QoL assessment. All evaluation data was recorded prior to the trial and at the eighth week of interventions for both groups.

**Results:**

All participants achieved improvements in shoulder ROM, muscle power, pain, and all aspects of QoL; however, higher statistical improvements were reported in all measurements with respect to strengthening exercises group (*p* < 0.001).

**Conclusion:**

The addition of graded Thera-Band strengthening exercises and scapular stabilization exercises in post-mastectomy AC rehabilitation program has significant benefits in shoulder function and patients’ QoL.

Trial registration: This study is retrospectively registered at ClinicalTrials.gov NCT05311839.

**Supplementary Information:**

The online version contains supplementary material available at 10.1007/s00520-023-07641-6.

## Introduction

Breast cancer surgeries particularly mastectomy results in limited shoulder movement which can lead to arm, shoulder pain, and stiffness [1]. Females who underwent mastectomy have reported a significantly higher incidence of shoulder morbidity (17%) [2]. Shoulder joint connective tissue fibrosis is common in post-mastectomy patients [3]. One of the most common symptoms of upper extremity morbidities is restriction of shoulder joint range of motion (ROM) which is linked to a lower quality of life (QoL) [4].

Adhesive capsulitis (AC) or “frozen shoulder” is an insidious inflammatory disorder characterized by a painful, progressive decrease in passive or active glenohumeral joint ROM caused by gradual fibrosis and subsequent contracture of the glenoid capsule [5]. AC frequently progresses through 3 different phases; the first one is the painful freezing phase, remains from 2 to 9 months, and is associated with emergence of sharp, diffuse shoulder discomfort that usually aggravates during night; the second phase is the frozen phase, lasts from 4 to 12 months, where pain starts to fade with a gradual decrease in glenohumeral joint ROM; and the last phase is the thawing phase where there is a gradual regain of ROM and takes from 5 months to 2 years to complete [6, 7].

AC can be treated either conservatively or surgically. Conservative treatment consists of a variety of exercise techniques and physical therapy modalities such as hot–cold therapy [8], transcutaneous electrical nerve stimulation (TENS), ultrasound (US), acupuncture [9, 10], and laser [11]. Active and passive ROM exercises, self-stretching, stretching exercises under the guidance of a physiotherapist, mobilization and manipulation techniques, resistance exercises, patient education, and home exercises are all part of the exercise program [12].

Exercise therapy might help in the reduction of pain and restoration of the range, coordination, and control of movement in patients with AC [13]. Graded resistance exercises are effective in decreasing fatigue levels and enhancing functional capacity and muscle strength [14]. Progressive strengthening exercise is extremely effective in reducing sarcopenia. Strength training improves muscle strength by increasing muscle mass and enhancing the recruitment and the firing rate of motor units [15]. Thera-Bands are a type of resistance exercise training tools which can provide a variable resistance and allow changes over ROM, thus preventing the risk of higher weight loading during strengthening exercises. In addition, elastic bands can provide efficient resistance and enhance muscle activation for promoting muscle strength in treatment of shoulder diseases. They are frequently used during exercise rehabilitation programs due to their simplicity, economic, and safety benefits [16–18].

The scapular stabilization exercise can be applied to patients with limited shoulder joint mobility and functional deterioration such as shoulder impingement syndrome, AC, and rotator cuff injury. As a result, rehabilitation exercises which strengthen the scapula’s stability can be extremely beneficial in rehabilitation treatment of patients with shoulder pain and problems [19].

Since the incidence of shoulder morbidity is six times higher following mastectomy compared to conservative therapy [20], which in turn influencing the patients’ ability on performing ADL, in addition to the lack of literature research on AC rehabilitation following mastectomy, the need of developing an efficient exercises program is necessary; hence, this experiment targeted to assess the impact of combining Thera-Band strengthening exercises with scapular stabilization training on AC following mastectomy in term of shoulder pain, function, and QoL.

## Methods

### Design and setting

This study was a randomized controlled experimental trial and was approved by the Ethical Committee of Faculty of Physical Therapy, Cairo University. The study was retrospectively registered in the Clinical Trials Registry (No: NCT05311839), and it was carried out between January 2021 and December 2021 at the outpatient clinic of Faculty of Physical Therapy, Cairo University. All the procedures used were conducted following the ethical rules of the Declaration of Helsinki.

### Participants

Seventy females diagnosed with one-sided post-mastectomy AC participated in this study. All patients were diagnosed by a specialized orthopedist. The procedures of this trial were carried out at the outpatient clinic at the Faculty of Physical Therapy, Cairo University. Females who met the following inclusion criteria participated in the study; age ranged from 40 to 60 years, ^2nd^ phase of AC, shoulder pain and stiffness for at least 3 months, and restriction in shoulder ROM (involving flexion, abduction, and internal/external rotation) less than 50% when compared to the other shoulder. The exclusion criteria included shoulder or acromioclavicular joint osteoarthritis, bone diseases, infection, severe osteoporosis, tumors or metastasis, history of previous shoulder trauma or accidental injuries, previous history of dislocation, surgery on the specific shoulder, and any other shoulder problems as supraspinatus tendonitis and impingement, neurological diseases (parkinsonism, stroke, radiating pain to arm), recent shoulder fracture or wound, diabetes mellitus, rheumatoid arthritis, and severe psychiatric illness.

Prior to participating in the trial, all participants were educated about the trial’s purposes, benefits, and steps and signed a consent form. Small groups of one to four patients underwent the sessions at a time, supervised by specialized physical therapist. The study included two equal groups of participants who were allocated randomly. The intervention group received graded Thera-Band exercises for the restricted shoulder ROM (flexion, abduction, and internal/external rotation) and scapular stabilization exercises (60 min, 5 days per week for 8 weeks) plus a conventional physiotherapy program including hot packs, active ROM exercises, pendular exercises, wall climb exercises, mobilization exercises, and shoulder capsular stretching. The control group obtained only the conventional physiotherapy program (30–40 min, 5 days weekly for 8 weeks).

### Sample size and randomization

The estimated sample size for this trial was 32 participants for each group, as calculated using G*POWER statistical software (version 3.1.9.2) according to shoulder external rotation data from a pilot study conducted on 5 subjects per group. α = 0.05, power is 80%, effect size = 0.72, and allocation ratio N2/N1 = 1 was used in the calculations. For a possible drop out, the sample size was increased by 10%. Randomized assignment of patients was done equally either to intervention group (*n* = 35) or control group (*n* = 35)**.** The randomization process was carried out by a physiotherapist who was not involved in the data collection processes. Each patient received a unique computer-generated random code using the GraphPad software© (1:1 simple randomization), concealed in a sealed envelope. The sealed envelopes were given to the physiotherapist before the treatment. The results were collected prior to the trial and at the 8th week of therapy by therapist who was blind to the distribution process.

### Treatment procedures

All patients within both groups received the conventional physiotherapy program, 5 days a week for 8 weeks, and the duration of treatment was approximately 30–40 min.

#### The control group

The participants within this group received the conventional physiotherapy program including hot packs (5–10 min), passive mobilization exercises for glenohumeral (GH) joint, and scapulothoracic articulation. GH joint mobilizations (posterior gliding for increasing flexion and internal rotation, caudal glide for increasing abduction, and anterior gliding for increasing external rotation). Scapulothoracic mobilization was applied for improving the movements of scapula (protraction/retraction, elevation/depression, and rotation). For active ROM exercises and pendular exercises, the patient was instructed to lean forward and place the unaffected hand on a table. While keeping a straight back and a relaxed shoulder, softly sway the arm forth and backward; the exercise was repeated by shifting the arm from side to side and then in an orbicular movement (10 reps, 5 times per day), wall climb exercises (hold for 15–30 s at the peak, 10 reps, 5 times daily), and capsular stretching exercises (anterior, posterior and inferior capsular stretches) and sustaining for 15–30 s, 5 reps, 5 times/day [21, 22].

#### The intervention group

The participants within this group received graded Thera-Band exercises and scapular stabilization exercises (60 min, 5 days per week for 8 weeks) plus a conventional physiotherapy program.

##### Thera-Band exercises

Patients in the intervention group received graded Thera-Band exercises (Thera-Band®, Hygenic Corporation, Akron, OH, USA). For Thera-Band application for shoulder flexors and abductors, the patient was in a comfortable standing position with both feet firmly positioned on the Thera-Band. The patient was instructed to grasp the end of the Thera-Band and gradually flex and abduct the shoulder from the starting position, hold for (25 s), and then return to the starting position without bouncing. For shoulder internal rotation, the patient was asked to stand and hold the Thera-Band in the hand while the direction of resistive power away from the side at the level of elbow which is bent to 90°. The patient was instructed to internally rotate the arm by pulling across the front of the trunk. For shoulder external rotation, the patient was instructed to stand with Thera-Band beside the body at level of elbow and to flex her elbow to 90°, grasping the elastic band and rotating the arm laterally. All patients performed the above exercises for 30 min with 2–3 series of 10–15 reps of every exercise. All patients started strengthening exercises with the yellow color, progressed to the red, and then the green. When the patient could easily complete three sets of 10–15 reps, progression to the next color was considered [21–23].

##### Scapular stabilization exercises

In addition to graded Thera-Band exercises, the patients in the intervention group performed the following scapular stabilization exercises for 30 min, 10 reps each:

1. Scapular clock exercises: in the standing position, the patient was asked to place the arm on a wall with fully extended elbow, with the fingers directed towards the 12, 3, 6, and 9 o’clock positions. These exercises improved scapular elevation, protraction, depression, and retraction, respectively.
2. Towel slide exercises: with slightly flexed elbow, the patient was instructed to place the hand on a towel on the wall and wash the wall in approximately a 12 inch backward and forward motion, moving from the extended arm, retracted scapula, and to a flexed arm and protracted scapula.
3. Ball stabilization exercises: while standing close to the wall, the participant was asked to position her affected hand on the ball and keep the ball from moving as disturbance was applied in different directions.
4. Lawnmower exercises: the patient stood with abducted legs, bent knees, and holding a weight in the hand for resistance. The patient was asked to pull using large amounts of lower extremity extension and trunk rotation to guide the shoulder movement.
5. Serratus anterior punch: in standing position, the patient was instructed to perform alternative serratus anterior punches while holding the Thera-Band for resistance [24, 25].

### Outcome measurements

Physical function, pain, and QoL were among the outcome measures. The measures were taken before the trial and at the end of the eighth week of therapy. Shoulder range of motion, muscles strength, and the Disability of the Arm, Shoulder, and Hand questionnaire (DASH) are all used to measure physical function. Shoulder pain was assessed by visual analogue scale (VAS). The Medical Outcomes Study short-form questionnaire (SF-36) was used to assess QoL.

A digital goniometer with good reliability (*r* > 0.84) was utilized to quantify shoulder flexion and abduction ROM in supine with extended elbow, while internal/external rotations were measured in sitting with adducted shoulder and mid position of forearm. All measures were taken 3 times, and then the average was scored [26, 27].

A handheld dynamometer (J Tech Commender Muscle Tester, Salt Lake City, UT, USA), a valid tool (*r* = 0.81), was employed to test the muscle power of shoulder flexors, abductors, and internal/external rotators by measuring maximum isometric contraction in kg. Each record was done 3 times, and then the average was scored [28].

The DASH was designed to evaluate upper limb disorders and impairment and track changes and functional level over time. This questionnaire’s Arabic version is regarded as a plain, reliable (*r* = 0.97), and validated (*r* = 0.94) measurement instrument. The more the score, the more severe the symptoms [29].

The VAS is a reliable measure with an ICC of 0.97, consists of 10-cm line, and was utilized to quantify the pain severity within shoulder, where the score of zero means no pain, while a score of ten means significant pain [30].

The short-form SF-36, including physical function, role physical, general health, vitality, bodily pain, mental health, role emotional, and social function, was used for assessment of patients’ QoL. The Cronbach’s alpha coefficient was 0.94, while the inter-rater reliability was outstanding (ICC = 0.98) [31].

### Statistical analysis

An unpaired *t* test was employed to compare subjects’ characteristics between the groups. The chi-squared test was used to compare the allocation of the afflicted arm and adjunctive therapy between the groups. For ensuring normal data allocation, the Shapiro–Wilk test was utilized. For group homogeneity determination, Levene’s test for homogeneity of variances was utilized. The effect of therapy on VAS, DASH, shoulder ROM and strength, and QoL was investigated using a mixed model MANOVA. Post hoc test utilizing Bonferroni correction was performed for subsequent various comparisons. All statistical measurements had a significant level of *p* 0.05. For all statistical analysis, IBM SPSS (Chicago, IL, USA) version 25 for Windows (IBM SPSS, Chicago, IL, USA) was used.

## Results

### Participants’ characteristics

**Fig. 1:**
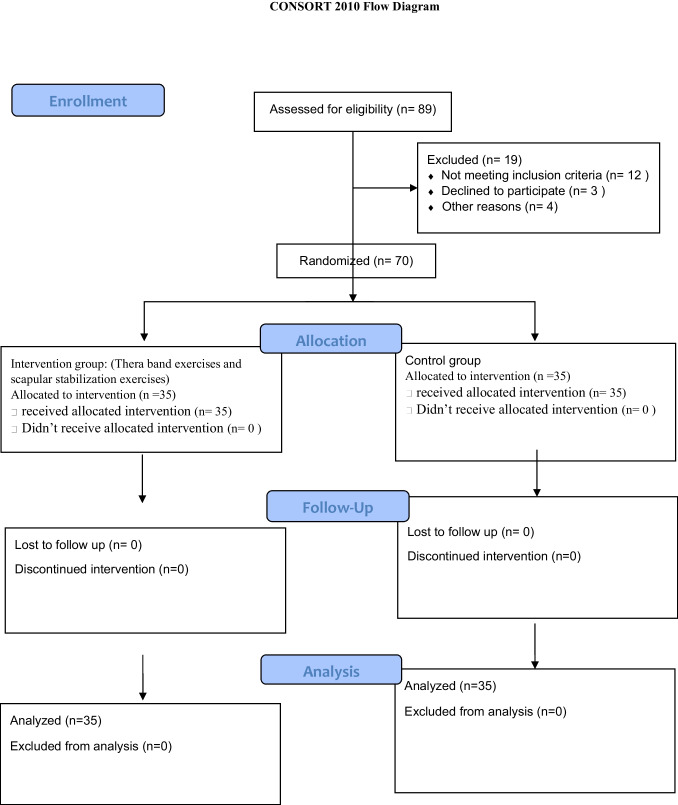
The patients’ flow diagram through the trial

The patients’ flow diagram through the trial is illustrated in Fig. 1. Seventy patients participated in this study with no significant difference in subjects’ age, BMI, affected arm, and adjunctive therapy distribution between the groups (*p* > 0.05) (Table 1).

**Table 1:** Participants’ characteristics

	Intervention group	Control group	*p* value
Age, mean ± SD (years)	49.54 ± 5.33	50.57 ± 4.69	0.39
BMI, mean ± SD (kg/m^2^)	27.43 ± 1.44	27.61 ± 1.07	0.54
Time since surgery, mean ± SD (month)	12.57 ± 1.68	13.05 ± 1.59	0.21
Affected arm, *n* (%)			
Right	22 (62.9%)	19 (54.3%)	0.46
Left	13 (37.1%)	16 (45.7%)	
Adjunctive therapy, *n* (%)			
Radiotherapy	22 (62.9%)	24 (68.6%)	0.61
Chemotherapy	25 (71.4%)	21 (60%)	0.31
Hormonal	10 (28.6%)	12 (34.3%)	0.61

*SD*, standard deviation; *p* value, probability value

### Intervention effect on VAS, DASH, shoulder ROM, and strength and QoL

The interaction of intervention and time was significant (*F* (18,51) = 139.81, *p* = 0.001, = 0.98). The main effect of time was significant (*F* (18,51) = 1181.78, *p* = 0.001, = 0.99). The main effect of intervention was significant (*F* (18,51) = 35.59,* p* = 0.001, = 0.92).

### Comparisons in each group

The VAS and DASH of both groups were statistically lower following intervention contrast to pre-intervention (p 0.001) (Table 2). The post-therapy shoulder ROM (flexion, abduction, external and internal rotation) and shoulder strength (flexors, abductors, internal/external rotators) of both groups were statistically higher than the pre-therapy levels (*p* 0.001) (Tables 3 and 4). Both groups demonstrated a significant improvement in all aspects of their QoL after intervention compared to pre-intervention (*p* < 0.001) (Table 5).

**Table 2:** Mean VAS and DASH pre- and post-treatment of both groups

	Intervention group	Control group		
	Mean ± SD	Mean ± SD	MD	*p* value
VAS				
Pre-treatment	6.88 ± 1.45	7.11 ± 1.56	-0.23	0.52
Post-treatment	2.54 ± 1.03	4.57 ± 1.39	-2.03	0.001
MD (% of change)	4.34 (63.08%)	2.54 (35.72%)		
	*p* = *0.001*	*p* = *0.001*		
DASH				
Pre-treatment	43.88 ± 6.59	44.62 ± 6.09	-0.74	0.62
Post-treatment	18.85 ± 4.22	30.17 ± 5.51	-11.32	0.001
MD (% of change)	25.03 (57.04%)	14.45 (32.38%)		
	*p* = *0.001*	*p* = *0.001*		

*SD*, standard deviation; *MD*, mean difference; *p* value, probability value

**Table 3:** Mean shoulder ROM pre- and post-treatment of both groups

ROM (degrees)	Intervention group	Control group		
	Mean ± SD	Mean ± SD	MD	*p* value
Flexion				
Pre-treatment	100.94 ± 10.74	98.97 ± 11.2	1.97	0.45
Post-treatment	162.37 ± 11.54	135.22 ± 9.88	27.15	0.001
MD (% of change)	− 61.43 (60.86%)	− 36.25 (36.63%)		
	*p* = *0.001*	*p* = *0.001*		
Abduction				
Pre-treatment	80.4 ± 9.22	79.8 ± 9.34	0.6	0.78
Post-treatment	128.48 ± 9.16	109.4 ± 10.23	19.08	0.001
MD (% of change)	− 48.08 (59.8)	− 29.6 (37.09)		
	*p* = *0.001*	*p* = *0.001*		
External rotation				
Pre-treatment	39.51 ± 6.57	38.6 ± 5.66	0.91	0.53
Post-treatment	68.77 ± 8.46	55.68 ± 6.6	13.09	0.001
MD (% of change)	− 29.26 (74.06%)	− 17.08 (44.25%)		
	*p* = *0.001*	*p* = *0.001*		
Internal rotation				
Pre-treatment	46.2 ± 4.82	45.88 ± 4.85	0.32	0.78
Post-treatment	75.4 ± 4.98	61.4 ± 5.83	14	0.001
MD (% of change)	− 29.2 (63.2%)	− 15.52 (33.83%)		
	*p* = *0.001*	*p* = *0.001*		

*SD*, standard deviation; *MD*, mean difference; *p* value, probability value

**Table 4:** Mean shoulder strength pre- and post-treatment of both groups

Strength (kg)	Intervention group	Control group		
	Mean ± SD	Mean ± SD	MD	*p* value
Flexors				
Pre-treatment	7.17 ± 1.15	6.97 ± 1.2	0.2	0.47
Post-treatment	11.94 ± 1.47	8.91 ± 1.01	3.03	0.001
MD (% of change)	− 4.77 (66.53%)	− 1.94 (27.83%)		
	*p* = *0.001*	*p* = *0.001*		
Abductors				
Pre-treatment	6.88 ± 1.05	6.57 ± 0.91	0.31	0.18
Post-treatment	10.82 ± 1.29	8.34 ± 1.08	2.48	0.001
MD (% of change)	− 3.94 (57.27)	− 1.77 (26.94)		
	*p* = *0.001*	*p* = *0.001*		
External rotators				
Pre-treatment	7.02 ± 1.09	6.65 ± 1.13	0.37	0.16
Post-treatment	11.17 ± 1.2	8.28 ± 1.1	2.89	0.001
MD (% of change)	− 4.15 (59.12%)	− 1.63 (24.51%)		
	*p* = *0.001*	*p* = *0.001*		
Internal rotators				
Pre-treatment	6.6 ± 1.11	6.2 ± 1.13	0.4	0.14
Post-treatment	10.25 ± 0.95	7.8 ± 1.05	2.45	0.001
MD (% of change)	− 3.65 (55.3%)	− 1.6 (25.81%)		
	*p* = *0.001*	*p* = *0.001*		

*SD*, standard deviation; *MD*, mean difference; *p* value, probability value

**Table 5:** Mean pre- and post-treatment of quality of life of both groups

	Intervention group	Control group		
	Mean ± SD	Mean ± SD	MD	*p* value
Physical functioning				
Pre-treatment	49.82 ± 3.88	48.57 ± 3.94	1.25	0.18
Post-treatment	73.05 ± 5.72	60.6 ± 5.12	12.45	0.001
MD (% of change)	− 23.23 (46.63%)	− 12.03 (24.77%)		
	*p* = *0.001*	*p* = *0.001*		
Role physical				
Pre-treatment	47.57 ± 4.81	46.71 ± 4.83	0.86	0.46
Post-treatment	67.02 ± 5.81	57.74 ± 4.53	9.28	0.001
MD (% of change)	− 19.45 (40.89)	− 11.03 (23.61)		
	*p* = *0.001*	*p* = *0.001*		
Bodily pain				
Pre-treatment	46.48 ± 7.15	45.2 ± 6.6	1.28	0.43
Post-treatment	66.02 ± 8.66	56.45 ± 6.57	9.57	0.001
MD (% of change)	− 19.54 (42.04%)	− 11.25 (24.89%)		
	*p* = *0.001*	*p* = *0.001*		
General health				
Pre-treatment	44.17 ± 7.28	43.4 ± 7.27	0.77	0.65
Post-treatment	67.34 ± 5.88	55.14 ± 6.09	12.2	0.001
MD (% of change)	− 23.17 (52.46%)	− 11.74 (27.05%)		
	*p* = *0.001*	*p* = *0.001*		
Vitality				
Pre-treatment	45.62 ± 7.26	44.8 ± 6.95	0.82	0.62
Post-treatment	62.57 ± 7.08	52.88 ± 7.27	9.69	0.001
MD (% of change)	− 16.95 (37.15%)	− 8.08 (18.04%)		
	*p* = *0.001*	*p* = *0.001*		
Social health				
Pre-treatment	54.68 ± 6.39	53.82 ± 4.34	0.86	0.51
Post-treatment	73.48 ± 5.66	63.28 ± 4.97	10.2	0.001
MD (% of change)	− 18.8 (34.38%)	− 9.46 (17.58%)		
	*p* = *0.001*	*p* = *0.001*		
Emotional health				
Pre-treatment	46.68 ± 4.52	46.31 ± 4.31	0.37	0.72
Post-treatment	69.34 ± 5.51	57.51 ± 4.85	11.83	0.001
MD (% of change)	− 22.66 (48.54%)	− 11.2 (24.18%)		
	*p* = *0.001*	*p* = *0.001*		
Mental health				
Pre-treatment	43.4 ± 7.27	43.02 ± 6.61	0.38	0.82
Post-treatment	67.97 ± 6.55	54.17 ± 5.36	13.8	0.001
MD (% of change)	− 24.57 (56.61%)	− 11.15 (25.92%)		
	*p* = *0.001*	*p* = *0.001*		

*SD*, standard deviation; *MD*, mean difference; *p* value, probability value

### Comparisons between both groups

Pre-therapy, statistical difference between the groups was not reported (p > 0.05). Following therapy, the intervention group showed statistical lowering in VAS and DASH than the control group (*p* < 0.001) (Table 2). Moreover, comparing to the control group, the intervention group had higher statistical improvements in shoulder ROM (flexion, abduction, external/internal rotation), strength (flexors, abductors, external and internal rotators) (Tables 3 and 4), and in all aspects of QoL after therapy (*p* < 0.001) (Table 5).

## Discussion

Pain and ROM restriction within AC are likely due to fascial constraints, muscle stiffness with trigger spots, in addition to capsular and ligamentous rigidity [32]. This study was a randomized retrospective-controlled trial that aimed to assess the impacts of shoulder strengthening and scapular stabilizing exercises on physical function, pain, and QoL in post-mastectomy patients with AC, where the control group received 30–40 min of traditional therapy and the intervention group with both interventions received > 60 min. The results illustrated higher statistical improvements of all parameters in favor to the strengthening exercises group. All patients in this trial achieved improvement in pain and shoulder joint ROM, as mobilization has been showed to lower pain through the neurophysiologic impacts of mobilization on peripheral mechanoreceptor activation and nociceptors inhibition; additionally, improvement of ROM could be explained by the effect of mobilization on shoulder AC as posterior–anterior glide was chosen to improve the outer ROM, while caudal glide was chosen to improve abduction. It is possible that the glenohumeral joint’s posterior–anterior and caudal glides improved capsular extensibility and lengthened soft tissues, which were restricting joint motion. As a result of the greater capsular extensibility, the glenohumeral joint may have had more ROM. These treatments are also hypothesized to boost proprioceptive and kinesthetic sensations within the joint, allowing participants to perform tasks within their new ROM; as a result, the individual’s ROM can be maintained. Another possible explanation is the impact of stretching exercises which have been shown to improve the extensibility of soft tissue through the creep response, modifying viscoelastic characteristics and hence increasing ROM. Individuals must perform tasks within their newly acquired range of motion in order to retain joint motion. This conclusion backs up prior studies indicating that mobilization and stretching exercises can help with AC [33–36].

Following mastectomy, the main cause of shoulder dysfunction is not only the glenohumeral joint disorder but also the adhesions in the axillary and pectoral areas between the pectoral muscles, subcutaneous tissue, and skin that may prevent complete extension of the pectoralis, resulting in limitation of both shoulder flexion and abduction [37, 38]. So, introducing progressive strengthening exercises during treatment could overcome the consequence muscles weakness caused by adhesions and encourage gaining more shoulder range, promote neuromuscular control, ameliorate general strength, and facilitate optimal strength ratios of the rotator cuff and scapular rotator muscles, and hence facilitate rapid recovery from AC, and that may illustrate the higher statistical improvement in all shoulder ROM within the resistance exercises group compared to the other group which in turn facilitate the activities of daily living (ADL) and improve the shoulder function. These results support and come in line with other studies [21–23, 39–42] and emphasize on the prominent role of strengthening exercises in AC rehabilitation, as Harishkumar et al. (2017) [21] added Thera-Band strengthening exercises to traditional care in AC cases and evaluated the shoulder function and ROM after 3 weeks of intervention which showed higher statistical improvements than receiving traditional care only, while Rawat et al. (2016) [22] studied the impacts of gradual resistance exercises of rotator cuff muscles beside receiving TENS and shoulder joint mobilization techniques for 1 month and reported significant ameliorations in shoulder functional level, pain, muscle strength, and ROM. Datar and Devi [23] studied the effects of Thera-Band strengthening exercises on shoulder dysfunction following mastectomy in term of muscle power, shoulder functional capacities, and ADL; evaluations were done after 2 months of intervention and demonstrated that Thera-Band exercises have significantly improved the outcome measures for strength and daily tasks involving upper extremity.

Shrug sign is one of the issues that may be exhibited during application of resistance exercises for shoulder abduction. One of the benefits of Thera-Band is the ability to assist in rotator cuff retraining during starting abduction, as the elastic band generates “upward and inward” resistance vector against which the patient must push in a “downward and outward” direction. This action activates the start of abduction additionally the rotator cuff’s depression and stability activities, which occur before and during abduction. According to anecdotal evidence, this exercise helps patients with the shrug sign diminish early upper trapezius activity during abduction [32].

In terms of muscle strength, the resistance training group demonstrated better statistical results, which could be illustrated by the fact that strengthening exercises cause significant physiological variations in skeletal muscles including contractile and/or non-contractile muscle compositions. Mechanical stress causes disruption of myofibers and extracellular matrix, which stimulates protein synthesis, resulting in muscle growth by increment the sarcomeres number, which leads to an increase in pinnation angle and fascicle length and so muscle expansion [43].

Several studies have documented alteration of scapular motion in the mastectomy side which in turn affect the shoulder motion pattern and contribute to incidence of frozen shoulder [44, 45]; scapular stabilizers’ weakness causes a disruption in the scapula–humeral rhythm, resulting in shoulder dysfunction and micro-damage to the shoulder muscles, capsule, and ligaments. The scapula must rotate vertically, tilt posteriorly, and rotate externally during overhead exercises; scapular stabilizers’ weakness causes an imbalance of force coupling between the trapezius, serratus anterior, and rhomboids, resulting in downward rotation, anterior tilting, and internal rotation of the scapula during arm abduction. This fatigue-induced weakness deficit may have a detrimental influence on scapular posture and allow for increased lateral scapular gliding during functional activities [25], so scapular stabilization is an important part of exercises therapy for optimizing scapular alignment during upper extremity movement and for providing direct control of the scapular posture, allowing for proper length–tension ratios in the shoulder muscles. Hence, adding scapular stabilization exercises in the rehabilitation program is very essential and coincided with various studies that support the role of these exercises in normally restoring both shoulder and scapular balance and motion [24, 25, 46–49] and that could be explained as one of the factors that assist in shoulder AC improvement, as Kirthika et al. (2015) [24] and Gulwani (2020) [25] added scapular stabilization exercises to conventional care in cases with 2nd phase of AC and evaluated the shoulder function and ROM after 2 weeks of intervention and concluded that those exercises are beneficial for enhancing shoulder ROM and functional abilities; also, Yatheendra et al. (2015) [46] evaluated the impacts of combined scapular stabilization exercises and mobilization techniques on patients with AC for 4 weeks; the study comes to the conclusion that both interventions are effective in reducing shoulder discomfort, enhancing ROM, and improving functional capacity in AC.

Moreover, the experimental group demonstrated greater improvement in ADL regarding to the DASH scores and additionally in QoL which may be attributed to the crucial role of strengthening exercises among the various forms of physical exercise programs due to the link between muscle impairment, pain, and dysfunction [50], as strengthening of shoulder and scapular stabilizers has significant impacts in reducing pain and improving shoulder ROM, functional capacity, and muscle power by regaining scapula–humeral rhythm in AC [32].

The study confirmed the importance of exercises therapy in AC management without reporting any adverse effects and presents the preliminary evidences for introducing strengthening exercises as an essential part in AC rehabilitation; however, some limitations must be considered when explaining these results; the most significant drawback of this experiment was the lack of scapular movement analysis which could provide better statistical results; other restrictions are the absence of blinding during the treatment sessions and absence of AC imaging assessments that could provide better prognosis; also the long-term effect of treatment was not examined due to the difficulty of following up after the trial, so future trials analyzing the scapular motion with patients’ follow-up are recommended; also it is critical to promote knowledge about the protection, early diagnosis, and quick treatment of shoulder problems following mastectomy in order to reduce women sufferance and financial costs, so trials should be conducted to evaluate early physical therapy intervention in prevention shoulder morbidity following mastectomy; moreover, evaluation of the impact of different approaches of exercises therapy with longer duration should be carried out.

## Conclusion

Rehabilitation exercises program including progressive strengthening of both scapular and shoulder muscles played a significant role in improvements of shoulder ROM and function which reflected on patients’ QoL and ADL, so emphasis on strengthening exercises during rehabilitation can be of great benefit in AC treatment.

## Supplementary Information

Below is the link to the electronic supplementary material.

Supplementary file1 (DOC 219 KB)

## Data Availability

The data sets generated during and/or analyzed during the current study are available within the manuscript.

## Abbreviations

QoL: Quality of life
AC: Adhesive capsulitis
ROM: Range of motion
VAS: Visual analogue scale
DASH: The Disability of the Arm, Shoulder, and Hand questionnaire
SF-36: Short-form
